# Toxicological Effects of Technical Xylene Mixtures on the Female Reproductive System: A Systematic Review

**DOI:** 10.3390/toxics10050235

**Published:** 2022-05-04

**Authors:** Noor Asyikin Suaidi, Mohammed Abdullah Alshawsh, See-Ziau Hoe, Mohd Helmy Mokhtar, Siti Rosmani Md Zin

**Affiliations:** 1 Department of Anatomy, Faculty of Medicine, Universiti Malaya, Kuala Lumpur 50603, Malaysia; asyikin.suaidi@gmail.com; 2 Department of Pharmacology, Faculty of Medicine, Universiti Malaya, Kuala Lumpur 50603, Malaysia; alshaweshmam@um.edu.my; 3 Department of Physiology, Faculty of Medicine, Universiti Malaya, Kuala Lumpur 50603, Malaysia; hoesz@ummc.edu.my; 4 Department of Physiology, Faculty of Medicine, Universiti Kebangsaan Malaysia, Kuala Lumpur 56000, Malaysia; helmy@ukm.edu.my

**Keywords:** xylene, female reproductive system, reproductive toxicity, endocrine disruption, ovarian toxicity

## Abstract

Technical xylene is a compound of massive production that is used in applications such as petrochemical and healthcare laboratories. Exposure to xylene can cause acute and chronic effects in humans and animals. Currently available studies regarding xylene’s adverse effects with credible designs were dated almost twenty years ago. This systematic review summarizes the findings regarding the detrimental effects of technical xylene from human, animal, and in vitro studies. It recapitulated available studies with respect to the effects of xylene on the female reproductive system to stress the need for updating the current data and guidelines. Based on pre-specified criteria, 22 studies from journal databases exploring the toxic effects of xylene on menstruation, endocrine endpoints, fetal development, and reproductive functions were included for the review. It was found that related studies with a specific focus on the effects of technical xylene on the female reproductive system were insufficient. Therefore, further studies are necessary to update the existing data, thus improving the quality and reliability of risk assessment of exposure to xylene in pregnant women

## 1. Introduction

**Figure 1:**
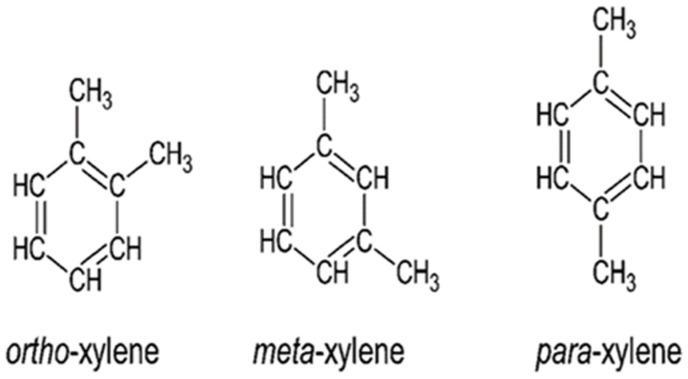
Xylene isomers.

Technical xylene, or simply referred to as xylene or xylol [1,2], is an organic chemical compound from the benzene family. With a chemical formula of C_8_H_10_ and a molecular weight of 106.2 g/mol, technical xylene is a mixture of three individual isomers (Figure 1), namely ortho-xylene (o-xylene), meta-xylene (m-xylene), and para-xylene (p-xylene) [1]. Being a cyclic aromatic hydrocarbon, xylene is a flammable, colorless liquid with a pleasant odor [1,3].

Xylene is produced through the extraction of crude oil and raw natural gas, where the compound is extracted from the reformulation of naphta during the petroleum refining process [1,4]. It is widely used in industries, household products, and healthcare laboratories. Approximately 30 million tons of xylene are used across the globe each year, and it is one of the most highly manufactured chemicals in the United States. It is broadly used as an organic solvent and chemical feedstock in manufacturing industries [1,2]. In healthcare settings, xylene is one of the major chemicals commonly used in histological laboratories. The analytical grade of xylene, with a purity of 99.7% of xylene constituents, is used by healthcare histology technicians and medical laboratory technologists for tissue processing, staining, and cover-slipping the stained slides in order to permanently preserve the tissue samples for further examinations by the pathologists [5]. Xylene acts as a clearing agent, with the purpose of making the histological tissue sections clear or transparent so that the detailed morphological structure of the tissues can be examined [6].

There are many published articles showing the evidence of the health effects of xylene as a constituent of organic solvents to both humans and animals. Previous studies have demonstrated that the individual isomers of xylene have almost similar toxicokinetic characteristics and toxicological effects [7]. The harmful effects of xylene have been investigated by several authors [2,3,4,5,6]. Exposure to xylene affects the central nervous system, respiratory system, and hepatic system. The adverse effects include increased dopamine in the brain, irritation of the nose and throat, and increased liver weight [3]. In relation to the reproductive effects of xylene, there were some deficiencies in the previously published studies regarding the duration of animal exposure to conclude the no-observed-adverse-effect level (NOAEL) [3,8,9]. In addition, reviews on the reproductive effects of technical xylene are still lacking with their focus on the female reproductive outcomes, morphologically and histologically. This systematic review will highlight the currently available studies assessing the detrimental effects of technical xylene exposure to the female reproductive system, despite the presumptions that data are growingly up to date.

## 2. Materials and Methods

This section describes the strategy applied to retrieve articles within the scope of the field of interest. The step-by-step review process was achieved with the aid of Preferred Reporting Items for Systematic Reviews and Meta-Analyses (PRISMA). The process also comprise the search of published articles within the key area of interest, which were sourced from several online databases. Later, the searched articles were narrowed down based on the inclusion and exclusion criteria. Through a detailed systematic review, the final retrieval of study articles was accomplished for data mining and analyses.

### 2.1. PRISMA

PRISMA is frequently adopted within medical research and health care [10] because of its ability to identify the inclusion and exclusion criteria [11]. On top of that, PRISMA allows systematic searches by identifying clear research questions. From a large database of scientific literature, it can examine the records within a specified time [12]. Due to these advantages, PRISMA was a useful tool to accurately gather the articles and to identify the current studies investigating the toxicity of technical xylene, particularly on the female reproductive system.

### 2.2. Search Strategy

Research articles for the review were resourced from six journal databases: Web of Science (WoS), Scopus, Science Direct, PubMed, Dimension, and Google Scholar. The use of Dimension and Google Scholar allowed both indexed and non-indexed studies to be retrieved, thus providing a comprehensive, non-biased result [13]. The systematic review process began with the identification of research articles with a high potential of being parallel to the objectives of the review. To achieve this, a search strategy was applied by identifying the relevant keywords and phrases depicting the subject of interest. Guided by previous studies to find all relevant articles, the search was further strengthened using the keywords’ similar term with the aid of a thesaurus, dictionaries, and the Medical Subject Headings (MeSH) function to target keywords such as “technical xylene exposure”, “xylene administration”, “xylol inhalation”, “mixed xylene” combined with “reproductive toxicity”, “developmental toxicity”, “detrimental effects”, “endocrine fate” and “female reproduction”. Table 1 shows the search string used for article searching from two major databases. A similar query or syntax was used for other databases.

**Figure 2:**
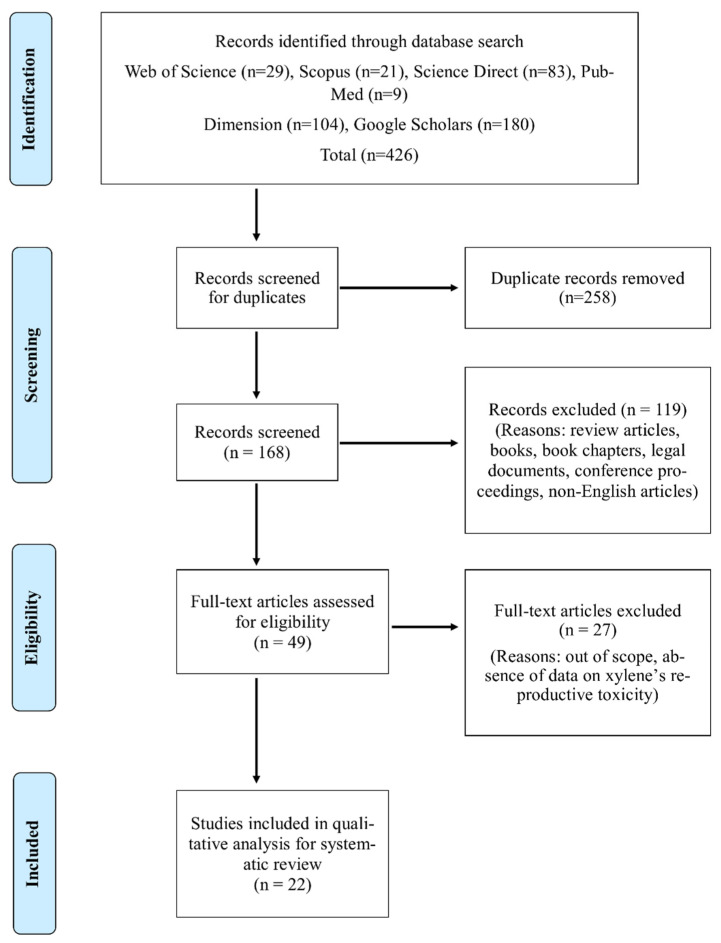
Article search and identification using PRISMA [14].

The next stage was the screening of all available identified articles. Accordingly, a total of 411 articles were obtained from a primary search of the previously stated databases. Aided by PRISMA, Figure 2 illustrates the step-by-step process of identifying and screening the articles eligible for the systematic review.

### 2.3. Inclusion and Exclusion Criteria

The criteria of selection and exclusion were determined for the review process according to population, exposure, comparator, and outcomes, or the PECO model [15]. Only research articles containing empirical data on the reproductive effects of technical xylene in women of reproductive age, pregnant animals, and ovarian cell lines were selected (Table 2).

Review articles, books or book chapters, legal documents, patents, and conference proceedings were excluded. Secondly, to avoid mistranslation and confusion with the non-English papers, e.g., Chinese and Arabic, only research articles published in English were included. Additionally, this review only included literature published within a 30-year timeline, which was from 1990 to 2022, to evaluate the trend of studies and publications in the key subject area. In terms of geographical eligibility, the review included research articles from across the globe. A summary of the inclusion and exclusion criteria is shown in Table 3.

### 2.4. Data Retrieval and Analysis

All remaining 22 research articles were thoroughly assessed based on their objectives, methodologies, and findings. Research papers regarding the toxicity of xylene compounds to the female reproductive system were categorized into in vitro studies, in vivo studies, and human studies.

## 3. Results

Through a rigorous search sourced from several databases, a total of 22 research articles, highlighting the detrimental effects of xylene on female reproduction, were obtained. These articles were divided into three major categories based on their methodologies, namely in vitro studies, in vivo studies, and human studies, as summarized by Table 4, Table 5 and Table 6.

### 3.1. In Vitro and In Vivo Studies

Studies were carried out on ovarian cell lines to investigate the effect of xylene on different reproductive parameters, such as cellular replications and sexual hormones. In addition, in vivo studies pertaining to the toxicity secondary to xylene exposure on the female reproductive system were conducted using different animal models. The xylene compounds used for these studies were either xylene alone or as a mixture with other organic solvents.

#### 3.1.1. Ovarian Cell Toxicity

Disturbances in maternal ovarian functions have been studied as part of the embryotoxic mechanisms of xylene during pregnancy [37], as the mammalian ovarian gland is the ultimate point for the sympathetic and sensory neurons responsible for the establishment and maintenance of ovarian functions [38]. Xylene had demonstrated to be hindering the basic cellular functions of ovaries, namely cellular replication, hormone production, and apoptotic activities. Such findings were discovered by Tarko et al. [18] through in vitro studies using Holstein cows’ ovarian granulosa cell culture. Via immunoassay and immunocytochemical analysis, they found that in vitro exposure of ovarian cells to xylene could interfere with the release of progesterone and testosterone. Simultaneously, xylene increased cellular multiplication and insulin growth factor 1 (IGF-1) production. The latter suggested that xylene could exert cancerous change characterized by amplified cellular replication [17,18].

Using the cultured porcine ovarian granulosa cells, Sirotkin et al. [19] found that xylene stimulated the build-up of proliferating cell nuclear antigen (PCNA), an indication of increased cellular replication. The result was found to be contrary in their later study, where xylene reduced the PCNA expression [21]. A xylene compound also surged the apoptotic activity, as shown by an elevated apoptotic marker, Bax, thus reducing the cellular viability. The levels of progesterone and oxytocin were also elevated following exposure of the cell cultures to 0.1% of xylene [19]. These findings also proved that the cultured ovarian cells are suitable to be used to assess the ovarian responses to external stressors. It was evident by the ability of cells to eliminate the Trypan blue and the presence of proliferation- and apoptosis-related compounds [19]. In another study, the team established that xylene, as part of BTEX, could induce apoptosis in porcine and bovine ovarian cell cultures, as indicated by the elevation of apoptotic markers, namely Bax and p53 [16]. They further investigated the adverse effects of xylene by comparing the expression of apoptotic and proliferation markers produced by ovarian cells harvested from cows of different body condition scores. They found that ovarian cells from cows having a higher body condition score were more resistant to the harmful effects of xylene compared to emaciated cows [20]. These in vitro studies provided useful data regarding the notorious effects of xylene on female reproductive functions by directly exposing the target cells to the compound.

#### 3.1.2. Maternal Toxicity

Xylene and its isomers had been found to cause a significant decrease in the maternal body weight and food consumption at a concentration of 1000 parts per million [9]. The study, however, did not reflect an occupational setting due to the continuous daily exposure of technical xylene to the tested female rats [9]. Inhalation daily exposure to technical xylene at 200 ppm during the organogenesis period did not cause maternal toxicity [22].

#### 3.1.3. Developmental Toxicity

Due to its lipid solubility characteristic, xylene is able to reach the embryos from maternal exposure through placental transfer [39,40]. Xylene and its isomers had been found to cause a significant decrease in fetal body weight at a minimum concentration of 500 parts per million [9]. Adverse fetal effects were manifested as malformations such as skeletal variations, visceral deformities, and abnormal ossification [9,22]. Saillenfait’s research was designed in accordance with OECD guidelines for the female reproductive system toxicity study (OECD, 2001) [41]. In different studies, the compound, as a mixture with other toxic solvents, e.g., methyl ketone and toluene, caused developmental malformations such as diaphragmatic hernia, absence of tail, and skeletal variations. These studies, however, were weakly associated with a xylene compound [23,24].

Prenatal inhalation exposure to xylene, as part of the thinner mixture during the whole gestational period of pregnant mice, was also found to cause developmental toxicity and behavioral changes in mice offspring, according to Malloul’s study [26]. Significant adverse effects were observed following prenatal exposure to paint thinner and other xylene-containing solvent mixtures at 100 ppm to 2000 ppm. Several notable effects include shortened offspring’s body length, retarded sensorimotor development, anxiety, and depression [26,42]. Her study emphasized the inhalation exposure that mimicked inhaled solvent abuse (thinner sniffing) among pregnant women, rather than exposure from an occupational setting.

Singh et al. [25] tested the potential cellular toxicity of xylene, benzene, and toluene on *Drosophila melanogaster* (*D. melanogaster*) larvae and found that these compounds induced heat shock proteins and the reactive oxygen species (ROS), which subsequently delayed the development of adult flies for 48 h in the xylene-treated groups. In addition, xylene caused a significant decrease in the number of eggs laid by female *D. melanogaster* [25].

### 3.2. Human Studies

The associations between xylene exposure and female reproductive functions in humans were investigated through cross-sectional population studies. None of this research was conducted with the specificity of investigating xylene alone as the compound of interest, but rather as a mixture of organic solvents or volatile organic compounds (VOCs). Nevertheless, there is a high possibility of interaction between xylene and other organic solvents due to its ability to act as inducer or inhibitor of the microsomal cytochrome P450 enzymes in the liver. Interactions of xylene with other compounds are challenging due to differences in metabolism among the exposed species [7]. The effect of xylene and alcohol mixture was, however, extensively studied, and it was found that alcohol consumption may inhibit xylene metabolism due to competition for mixed oxidases in the liver [7]. Other factors that contribute to xylene metabolisms and its effects include exercises, environmental factors, and pathological predispositions [43].

#### 3.2.1. Menstrual Disturbances

The female menstrual cycle has been accounted for as a vital parameter in assessing reproductive health [27,44]. Alteration of menstrual patterns is an indicator that can be used as a prediction of the internal state of the endocrine function, which may be linked to other chronic diseases such as breast cancer [44]. Merklinger-Gruchala et al. [45] used the menstrual cycle characteristics as a prognostic factor of women’s reproductive health. They stated that any disturbances in the menstrual cycle may hinder the oocyte quality, ovulation process, fertilization, implantation, or the fate of embryos [45].

The most recent study was carried out by Moradi et al. [33] using a cross-sectional population study. They investigated the occupational exposure of xylene, together with other compounds of benzene, toluene, ethylbenzene, and xylene (BTEX), among 36 beauty salon workers in the main city of Tehran [33]. They measured both salon indoor and outdoor BTEX levels using the National Institute of Occupational Safety and Health (NIOSH) 1501 method [46]. Urine BTEX metabolites collected from the beauty salon workers were also quantified using the headspace solid-phase micro extraction (HS-SPME) coupled with gas chromatography-mass spectrometry. The analysis results were used to correlate the BTEX exposure with menstrual disturbance indices, namely menstrual cycle length and menstrual flow. The urinary concentrations of m-xylene and o-xylene metabolites were between 99.3 ng and 276.3 ng/L and 57.6 ng/L and 149.2 ng/L, respectively. It was concluded that m-xylene and o-xylene caused menstrual disturbances, with a prevalence of 47% among beauty salons workers, which might be caused by day-to-day salon activities such as hair coloring, nail treatment, and hair removal [33,47]. Nevertheless, the threshold limit value (TLV) or the permissible exposure limit (PEL) for individual compounds in this study was not suitable when applied to non-industrial workplaces due to shorter exposure duration and inadequate protective equipment [33].

Using the same study approach, another research team studied the relationship between occupational exposure to xylene, as a mixture of four organic solvents (xylene, benzene, styrene, and toluene), and menstrual cycle length among 1408 female petrochemical workers in China [27]. Applying the logistic regression, they scrutinized the correlation between the low-level exposure of xylene, with other organic solvents and prolonged menstrual cycle length, or medically termed as oligomenorrhea. Oligomenorrhea was defined, for the study, as an average cycle length longer than 35 days. This also included a menstrual cycle lasting over 90 days [44,48,49]. The assessment was made through an interviewer-delivered questionnaire to the enrolled subjects. The study concluded that the exposure of xylene alone, at a low level, was associated with a prolonged menstrual cycle in 284 enrolled females, accounting for 14.1% of the total subjects. Although this study claimed that the exposure was at a low level, there were no quantitative analyses conducted to present the data with accurate values. The inference was made based on a report provided by an industrial hygienist in the research team [27].

#### 3.2.2. Endocrine Disruption

Endocrine disruptors are defined as exogenous agents that hinder the normal regulations of natural hormones that are responsible for the preservation of homeostasis and the developmental process [50,51]. Endocrine characteristics, particularly hormonal variables, are associated with female fecundability and, thus, fertility [52]. Studies have proven that reproductive hormonal quality may predict the possibility of pregnancy in women within their ovulatory menstrual cycle [28,52]. In 2002, a cross-sectional population study was conducted by Reutman and their team to explore the potential endocrine reproductive effects of mixed xylene exposures amongst female United States Air Force (USAF) employees. This study, however, did not investigate xylene, per se, but as a combination of solvents and fuel [28]. Four urinary endocrine endpoints were used, namely follicular progesterone, mid-luteal progesterone, luteinizing hormone, and estradiol, based on an established algorithm to assess the reproductive endocrine characteristics [28,52,53]. It was evident, from this study, that the occupational exposure of m-xylene, p-xylene, and o-xylene within the aromatic hydrocarbons significantly reduced the pre-ovulatory luteinizing hormone (15.8 vs. 22.0 mIU LH/mg creatinine) and almost significantly lowered the mid-luteal progesterone. The data were gathered and analyzed from 100 eligible participants. Such disturbances in the regulation of reproductive endocrine may carry predictive values in women’s reproductive performance [28].

#### 3.2.3. Adverse Birth Outcomes and Other Potential Reproductive Health Risks

Adverse birth outcomes, such as oral clefts and neural tube defects (NTDs), have been associated with maternal exposure to hazardous air pollutants (HAP) [54,55,56]. Lupo et al. [30] conducted a case-control population study to determine whether maternal exposure to ambient levels of BTEX is linked to NTDs among offspring in Texas, United States. They collected 1108 birth defect data from the Texas Birth Defects Registry from January 1999 to December 2004. The study, however, concluded that there was no significant relationship between ambient levels of xylene and offspring suffering from NTDs [30]. Although the potential confounders and etiologic heterogeneity were identified to minimize the errors, a few limitations were identified. In relation to exposure assessment, the 1999 United States Environmental Protection Agency (USEPA) Assessment System for Population Exposure Nationwide (ASPEN) model might cause misclassification to the data collected after 1999 due to environmental changes, such as roadways and industrial emissions [30].

Studies by Ghosh et al. [31] and Serrano-Lomelin et al. [34] showed strong correlations between low birth weight and xylene-containing traffic pollutants, as well as industrial air pollutants. In Serrano-Lomelin’s study, a significant spatial association (OR, 1.16) was discovered between low birth weight and xylene-containing industrial air contaminants among 333,247 singleton births from different postal code regions in Canada from 2006 until 2012 [34]. Their study also found associations between these pollutants and other adverse birth outcomes, namely preterm birth and small for gestational age [34]. These findings were supported by Aguilera et al. [29] in their cohort study, using the data from 562 women exposed to BTEX and nitrogen dioxide (NO_2_). Their study found a negative association between BTEX and the biparietal diameter of the subjects’ fetuses when exposed from week 20 to week 22 [29]. Furthermore, Ghosh et al. [31] discovered higher chances of delivering low-birth-weight infants among mothers who were exposed to BTEX-containing traffic pollutants during the third trimester. They collected 8181 low-birth-weight data and 370,922 normal-birth-weight data from 1995 to 2006 [31]. In addition, evidence of an association between BTEX exposure and preterm newborns was discovered by Cassidy-Bushrow et al. [35]. They found that preterm births, along with other maternal factors such as maternal age and ethnicity, were strongly linked (OR > 1.00) with ambient BTEX exposure [35].

In 2017, Santiago et al. [32] presented two case reports of acquired chromosomal aberrations in female gas station workers with chronic exposure to BTEX. Both females had a history of miscarriage during the first half of their pregnancy. Fluorescence in situ Hybridization (FISH) revealed complex chromosomal rearrangements (CCR) involving the derivatization and insertion of chromosomal breakpoints that were positively associated with reproductive risk and early pregnancy loss [32]. They suggested that chromosomal aberrations may be an indication of chemosensitivity induced by environmental contaminants such as BTEX [32].

The long-standing concerns regarding the reproductive outcomes from exposure to organic solvents in general and xylene in specific has been influenced by the ubiquitous nature of these compounds. Humans were at risk of being exposed through environmental, occupational [27,33,57,58], and other sources, including commercialized consumer products. In the most recent study, Ding et al. [36] conducted a cross-sectional population study to establish an association between exposure to m-xylene and p-xylene, as part of the volatile organic compounds and the application of feminine hygiene products among American women in their reproductive age [36]. Trace levels of m-xylene and p-xylene were found among women who used vaginal douches for a period of six months. Ding et al. [36] admitted that the study had several drawbacks in terms of the reliability of the results. The cross-sectional data gathered for their study did not address other possible sources of volatile organic compounds. In addition, the population data used were retrospective in nature, which were dated from 2001 until 2004. Thus, the norms of feminine hygiene practices 15 years later might not be accurately reflected in misclassification and recall bias [36].

## 4. Discussion

**Figure 3:**
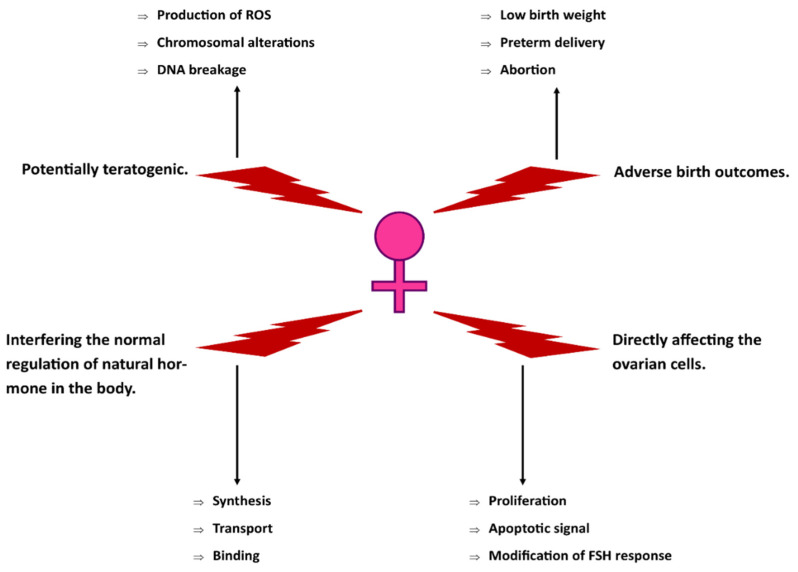
Suggested toxicity mechanisms of technical xylene on the female reproductive system.

This review paper was aimed to systematically assess the existing studies on the toxicity of xylene exposure to the female reproductive system. Nevertheless, technical xylene is also known to adversely affect the male reproductive system. Previously published studies showed that xylene caused histological alterations in the testes, such as increased intratubular spaces, reduced number of Leydig cells, and the degeneration of the spermatozoids in male rats [59]. In addition, a study on male rats reported that xylene may disturb the pubertal Leydig cell development by elevating the production of reactive oxygen species (ROS) [60]. Figure 3 highlights the proposed technical xylene’s mechanism of toxicity on the female reproductive system.

Most of the studies were conducted using animal and in vitro models to understand the effects of xylene on female reproductive functions. Two major areas were being focused on in the studies: developmental toxicities and ovarian cell toxicities. Developmental toxicities of xylene were scrutinized from the aspect of the maternal inhalation route. On the other hand, in vitro studies were the experimental approach to investigating ovarian toxicity resulting from xylene exposures. Literature that specifically highlighted the toxicity of technical xylene as an individual compound, not as a mixture with other organic solvents, were only available from animal studies [17,18,26].

Only one scholarly research article was identified regarding the female reproductive toxicity of technical xylene and its isomeric compounds of m-xylene, p-xylene, and o-xylene. Saillenfait et al. [9] studied the maternal inhalation exposure of technical xylene and its toxicity on rat development [9]. Maternal toxicities were reported as reduced body weight and declined food consumption at high levels of exposure (1000 ppm) from m-, p-, and o-xylene. In terms of scope and design, the study was considered comprehensive in exploring the maternal and developmental effects of technical xylene, since most of the previous studies were not tailored according to the current recommendations for chemical testing [41]. The study also managed to suggest the No Observed Adverse Effect Level (NOAEL) of technical xylene and its isomers for the rats and their offspring [9]. According to this study, the NOAELs of technical xylene and its isomers for maternal toxicity were between 100 ppm and 500 ppm [9]. Although continually cited in many research and review papers, as well as legal documents for many years as references related to xylene toxicity, there were gaps in this study suggestive of future research to address the unexplored variables. For instance, the maternal toxicities of xylene were only observed as decreases in weight and food consumption.

Due to their lipophilic properties, xylene isomers are quickly absorbed and distributed within the body, followed by the Cytochrome P450 (CYP450)-mediated oxidation to methylbenzyl alcohol, with urinary metabolites of 2-methylhippuric acid (2MHA), 3-methylhippuric acid (3MHA), and 4-methylhippuric acid (4MHA) from o-, m-, and p-xylene, respectively, of which they serve as an indicator for xylene exposure [7,61]. Much less is known about the exposure to mixed xylenes or the individual isomers and the populations at risk [62]. Based on earlier studies, oral administration of p-xylene at high concentration (2000 mg/kg/day) caused death in tested rats [63]. It was suggested that the biotransformation of p-xylene into a toxic tolualdehyde caused a 65% loss of microsomal hydroxylase activity [64]. The mechanism of how the technical xylene hampered the fetal development, resulting from maternal toxicity of xylene, was not thoroughly investigated. It was proposed by Miller and Edwards in 1999 that there were potential interactions between xylene isomers and the mechanism of their toxicities, which are yet to be revealed [65]. In terms of xylene exposure to the animal subjects, it did not mimic the occupational setting due to the constant daily exposure of xylene subjected to the rats, which was unlikely to happen in an occupational setting [9]. Furthermore, integrating animal data to establish the occupational exposure limits in humans involves several key uncertainties, such as interspecies variabilities and the establishment of safe levels from shorter duration studies to a lifetime exposure [66]. Inadequacies in the overall health effects database, where the most sensitive adverse effects are not investigated, also contribute to the insufficiencies [66]. To date, new studies to bridge these gaps remain insufficient.

Prenatal exposure to xylene, in the form of a mixture with other compounds such as thinner, has resulted in depression-like behaviors, impaired learning, and memory disturbance in mice’s offspring. Xylene, however, comprised only 15.5% of the product. Such a study was conducted to extrapolate the effects of prenatal and postnatal exposure to inhalant abuse among pregnant women to their babies [26]. However, the toxicity of xylene alone was not evaluated in these studies.

In vitro studies explored further the protective effects of certain vegetables and medicinal plants against xylene toxicity using bovine and porcine ovarian granulosa cells [17,18,21]. Nevertheless, the mechanism of xylene toxicity when absorbed into human and animal bodies is not well understood. It had been suggested that the biotransformation of xylene takes place in the liver through several enzymatic processes that produce a toxic aldehyde called p-tolualdehyde, which is believed to be the compound that exerts the toxic effects [65]. Preferably, the studies regarding the potential protective effects against xylene must be later verified by conducting suitable in vivo studies [18].

From in vitro studies using bovine ovarian cells, technical xylene was found to trigger the release of insulin-like growth factor hormone, IGF-1, and have the potential of inducing malignancy [17]. The study also found that xylene reduced the release of progesterone. Further investigation revealed that plant-derived flavonoids, called quercetin, and a medicinal plant, chia seeds, have protective effects against the deleterious action of xylene in vitro [17,18]. In contrast, other studies demonstrated stimulatory effects of xylene on the release of progesterone, while simultaneously hampering the cell survival indicated by an increase in apoptotic signal. These findings were evident through studies by Sirotkin and his team. Using a closely resembled experimental design, they assessed the in situ effects of xylene, as a mixture of environmental contaminants, on several mammalian ovarian cell cultures [16,19].

It is important that in vitro studies are validated through sufficient and reliable in vivo studies to reinforce the vital weaknesses of the non-physiological environment of in vitro cultures. It must be re-emphasized that the in vitro environment does not reflect the living animals’ body temperature, electrolyte concentrations, and metabolisms [18,67]. These in vitro studies also did not justify the selected concentrations of xylene used for the treatment as analogous to that of actual exposure to living animals. Furthermore, the scholars did not take into account that xylene is believed to affect female reproduction negatively through its metabolized aldehyde form, which occurs in the liver by microsomal enzymes [64,65]. In vitro tests were even, in some literature, referred to as impracticable for regulatory purposes [67]. Nevertheless, these scholarly articles have been useful in updating the currently available data related to the subject matter. The results could be potentially congruent to the readily documented data or future studies to explore in-depth the undesirable outcome of technical xylene to female reproductive functions.

From human studies, it was concluded that xylene affects menstruation by prolonging its cycle. This finding was evidenced through a couple of cross-sectional population studies aiming to explore the negative effects of occupational exposure of organic solvents and BTEX to female workers [27,33]. Oligomenorrhoea, following exposure to organic solvents at low levels, was also prevalent among the female employees of petroleum and chemical processing plants in China [27]. The occurrence of menstrual cycle disturbances among females following exposure to xylene was also probed within a smaller occupational setting. Additionally, the use of BTEX compounds in confined spaces such as beauty salons increased the ambient levels of xylene in the working space. Exposure to xylene in these smaller occupational settings was also found to cause menstrual disturbances with other health problems amongst female workers, e.g., skin irritations, headaches, and increased heart rate [33].

Apart from menstrual cycle disturbance, endocrine disruption was also found to occur following xylene exposure. It was evidenced from a study that occupational exposure to xylene and its isomers did affect the outcome of reproductive endocrine regulations [28]. In Reutman’s population study, several endocrine findings proved that xylene has an endocrine disruptive characteristic [28]. The endocrine functions of female employees working in the Air Force Base of the United States were assessed through questionnaires, interviews, and quantitation of urinary endocrine hormones, namely progesterone, luteinizing hormone, and estradiol. These endocrine landmarks were selected because of their predictive values of conception among women at their reproductive ages [28,52,53]. Although there have been reports on endocrine-related adverse outcomes (e.g., breast cancer and prostate cancer) of environmental pollutants, including xylene, scientific ambiguities remain on the cause of these reported effects [50]. Nevertheless, it has been suggested that endocrine is adversely affected by organic solvents and other environmental toxicants through hormone disruption where the chemical with a similar structure to endogenous molecules enters the reproductive organs and hampers the normal cellular processes, e.g., mitosis, meiosis, apoptosis, migration, and cellular signaling [68]. In agreement, environmental toxicants may induce cellular chemosensitivity, which leads to aberrant chromosomal alterations with an adverse outcome to female reproduction [32].

Due to the ubiquity of the source of exposure to the volatile organic compounds, there was an assessment conducted to evaluate the content of these chemicals in the feminine hygiene products being used by reproductive-aged women. Concerns regarding reproductive health were raised when feminine products such as vaginal douches, tampons, sanitary napkins, and feminine sprays were found to be the probable source of m-xylene and p-xylene exposures with other volatile organic compounds [36]. This recently published study, however, utilized the data regarding feminine hygiene products collected over a period of four years, more than 15 years ago. This study did not eliminate other contributing factors that might be responsible for compound exposure due to data absence. Furthermore, the nature of the cross-sectional data used for this study did not reflect the current pattern of the subjects being studied. In addition, the questionnaire, based on which the data were collected, was vulnerable to bias and inaccurate categorization [36].

The effects of xylene exposure on the female reproductive functions from human studies investigate only some of the xylene-containing organic solvents to which women are exposed in occupational settings. In addition, all studies were conducted as cross-sectional population studies, in which the study populations were selected based on pre-designed inclusion or exclusion criteria [27,28,33]. Due to the nature of occupational settings, from which the recruited subjects were exposed to the compounds of interest, none of these studies investigated technical xylene mixture in specific. Hence, it is highly recommended, as a future direction, that the reproductive effects of xylene should be studied as an individual compound, within an occupational setting such as the healthcare setting. In healthcare facilities, particularly histology processing laboratories, technical xylene is extensively used, not just as a pure compound but in massive volumes by laboratory personnel on a routine basis. This is in line with several published scholarly opinions that future studies must contribute in addition to the existing work and form the ground for another goal rather than reiterating the subject being studied [69,70].

Quoted from George E.P. Box, a renowned British statistician; “All models are wrong, some models are useful” [66], it should be taken into the fundamental understanding that scientific data from various study designs used in regulatory toxicology are subjected to limitations and drawbacks. Their core reliance on mechanistic models to reach any decisions remains unchanged significantly over decades; therefore, making animal studies the methods of choice in gathering toxicological data [67]. A huge number of toxicant-caused health problems are modeled by animals where their physiological states and tissues being tested, but a duplication of observed interactions are not attainable in non-animal models [67]. Human and animal (in vivo) studies and in vitro assays have been identified as the experimental approaches to fit the purpose of assessing the adverse effects of xylene on female reproductive outcomes. Many aspects of females’ reproduction were explored, i.e., menstrual regulation, ovarian functions, endocrine characteristics, as well as prenatal and postnatal developmental aspects [9,17,18,19,27,28,33,36,58].

In toxicology studies using animal models to determine the exposure limits, the toxicity responses are often assessed as a dichotomous response (e.g., presence or absence of malignancy), continuous measures (e.g., organ weight), or ordinal units (e.g., pain score) [71]. Although the dose-related effects are clearly defined, such studies are subject to remarkable uncertainties such as species differences, routes, and duration of exposure. Besides, the potency of the selected exposures in humans is highly unpredictable [71]. Generally, the occupational exposure limits can be established confidently by setting up a high-quality epidemiology study or an inhalation study using human volunteers under a fully regulated setting [72]. In chemical toxicity studies, both human and animal models pose challenges but are necessary to improve scientific certainty by fulfilling the requirements to integrate the chemical and species-specific data into the risk evaluation [72].

From the existing research articles taken for this systematic review, it can be summarized that the acquirement of data regarding the reproductive toxicity of xylene was vastly achieved through epidemiology and toxicology. Of these two disciplines, the latter provides more comprehensive knowledge pertaining to human exposures and adverse effects. None of these studies investigated xylene toxicities at the histological aspect of the female reproduction that could aid further understanding of xylene’s mechanism of toxicity. It was also stated that toxicological properties of xylene have not been thoroughly investigated [73]. Thus, these data must be kept updated with improved study approaches that are methodologically substantial to support and renew the existing information from previous literature.

Concerning occupational exposure to xylene, this compound was only studied as a joining member from a larger class of compounds, i.e., organic solvents, volatile organic compounds, or environmental pollutants [27,28,33]. There was no study found over the past 30 years that thoroughly investigated the effects of technical xylene as an individually used compound within occupational settings, except for the animal developmental toxicity study conducted by Saillenfait et al. [9]. In addition, the purpose of this review was to evaluate how much of the data regarding female reproductive and developmental toxicity of technical xylene has been updated. There were limited published studies that specifically focused on the toxicities of technical xylene to the female reproductive system. The process of incorporating toxicological science into human risk assessment is a complex effort. This review perhaps shall be able to provide justifications for future studies to fill the gaps of uncertainties regarding the adverse outcomes from xylene exposure to the female reproductive system. Furthermore, Langman [74] highlighted that the reported effects of xylene on pregnancy outcome and fertility in terms of spontaneous abortions and congenital defects are ambivalent [74].

## 5. Conclusions

This systematic review highlighted the current literature regarding the toxicity of technical xylene or mixed xylene on the female reproductive system. Based on human, animal, and in vitro studies, technical xylene affects the female reproductive system mainly from inhalational exposure. Technical xylene caused disturbances in menstrual regulation, endocrine performance, and ovarian cell functions. All human studies investigated the detrimental effects of xylene as organic solvent mixtures, which were appropriately in place according to the occupational settings of the studies. In the form of individual compounds, reproductive toxicities of xylene were only found from animal studies. Technical xylene exerts its toxicity to the female reproductive system possibly by interrupting hormone regulation or causing direct injuries to the ovarian cells (Figure 3). We recommend that future studies are necessary to clarify the uncertainties with regards to the toxicological effects of technical xylene exposure on the female reproductive system, both morphologically and functionally. Therefore, more relevant studies with improved study designs, methodologies, and scopes of research should be encouraged. In addition, further studies are needed to explore the mechanisms of the reproductive toxicity of xylene using appropriate animal models.

## Figures and Tables

**Table 1:** The search string. An asterisk (*) is used for multiple character searching.

Database	Search String
Web of Science	TS-((“xylene*” OR “xylol” OR “mixed xylene*” OR “dimethylbenzene” OR “xylene isomer*” OR “xylene toxicit*” OR “xylene exposure” OR “xylene administration” OR “xylene inhalation” OR “xylene treatment”) AND (“reproduct* system” OR ‘female reproduct*” OR “endocrine system” OR “menstrual regulat*” OR “ovar* function” OR “development*”) AND (“animal stud*” OR “human stud*” OR “in vitro stud*” OR “occupation* exposure*))
Scopus	TITLE-ABS-KEY((“xylene*” OR “xylol” OR “mixed xylene*” OR “dimethylbenzene” OR “xylene isomer*” OR “xylene toxicit*” OR “xylene exposure” OR “xylene administration” OR “xylene inhalation” OR “xylene treatment”) AND (“reproduct* system” OR ‘female reproduct*” OR “endocrine system” OR “menstrual regulat*” OR “ovar* function” OR “development*”) AND (“animal stud*” OR “human stud*” OR “in vitro stud*’ OR “occupation* exposure*))

**Table 2:** PECO (population, exposure, comparator, and outcome) model.

Variable	Description		
Population	Human population studies, animal experiments, and ovarian cells’ in vitro culture.		
	Human	Animal	In vitro cell culture
Exposure	Inhalation.	Inhalation.	Direct exposure to the target cells.
Comparator	Women of reproductive age with no exposure risks.	Animal receiving vehicle-only treatment.	Ovarian cells cultured without the test compound.
Outcomes	Menstrual disturbances, endocrine disruption, and adverse birth outcomes.	Decrease in the maternal body weight and food intake.	Increased cellular proliferation and apoptotic signals.

**Table 3:** Inclusion and exclusion criteria.

Criteria	Inclusion	Exclusion
Article category	Research articles (journals)	Systematic review journals, books or book chapters, legal documents, conference proceedings.
Language	English	Non-English
Duration of publications	From 1990 to 2022	Before year 1990
Geographical selection	Across the world	None

**Table 4:** In vitro studies on the toxicity of technical xylene to the female reproductive system from 1990 to 2022.

First Author (Year)	Location	Test Populations	Exposure	Organic Solvents Studied	Findings in Relations to Xylene
Sirotkin et al. [16]	Slovakia	Bovine and porcine ovaries’ granulosa cells.	Granulosa cells cultured with or without BTEX (1%).	BTEX.	Addition of 1% BTEX promoted apoptosis and cellular proliferation. Ovarian hormone (progesterone) increased significantly (16.38 ng/10^6^ cells/day vs. 7.64 ng/10^6^ cells/day).
Tarko et al. [17]	Slovakia	Ovarian cells of Holstein cows.	The ovarian granulosa were incubated with 20 ng/mL of 99% mixture of o-, m-, or p-xylene and chia seed extracts for 48 h.	o-, m-, and p-xylene.	Addition of xylene stimulated ovarian cell proliferation. It suppressed progesterone and testosterone release with or without the chia seed supplement.
Tarko et al. [18]	Slovakia	Ovarian granulosa cells of the Holstein breed.	The ovarian granulosa were incubated with a 20 µg/mL of 99% mixture of ortho-, meta-, or para-xylene and quercetin extracts (1, 10, or 100 µg/mL) for 48 h.	o-, m-, and p-xylene.	Addition of xylene stimulated ovarian cell proliferation and the release of IGF-1. It suppressed progesterone and testosterone release with or without the quercetin supplement.
Sirotkin et al. [19]	Slovakia	Porcine ovarian granulosa cells.	Ovarian granulosa cells were cultured with 0.1% xylene, toluene, or benzene for 2 days with and without porcine FSH (1, 10, or 100 ng/mL)	Xylene, toluene, and benzene.	Xylene and benzene (0.1%) stimulated the progesterone release, accumulation of PCNA, and apoptosis, thus reducing the cellular viability.
Sirotkin et al. [20]	Slovakia	Bovine ovarian cells from cows having different body condition scores.	Ovarian granulosa cells were cultured with 0.1% xylene, toluene, or benzene.	Xylene, benzene, toluene.	Xylene stimulated the expression of apoptosis and proliferation markers in the ovarian cells of cows having a low body condition score.
Sirotkin et al. [21]	Slovakia	Porcine ovarian granulosa cells.	Ovarian granulosa cells were cultured with 0, 10, 100, or 1000 ng/mL of xylene with 10 µg/mL of buckwheat, rooibos, or vitex plant extracts.	o-, m-, and p-xylene.	Xylene suppressed cell viability, induced apoptosis, and decreased the release of progesterone and estradiol. Addition of the studied plant extracts was able to mitigate the cellular toxicity from xylene.

BTEX, benzene toluene ethylbenzene xylene; FSH, follicle stimulating hormone; IGF-1, insulin-like growth factor; FSH, PCNA, proliferating cell nuclear antigen.

**Table 5:** In vivo studies on the toxicity of technical xylene to the female reproductive system from 1990 to 2022.

First Author (Year)	Location	Test Populations	Exposure	Organic Solvents Studied	Findings in Relations to Xylene
Hass and Jakobsen [22]	Denmark	72 pregnant Wistar rats. divided into two groups of 36 animals.	Inhalation exposure of 200 ppm of xylene or clean air from GD 4 until GD 20.	Technical xylene.	Exposure to 200 ppm of technical xylene from GD 4 until GD 20 did not exert maternal toxicity. However, the incidence of delayed ossification in the skull of the xylene exposed fetuses.
Saillenfait et al. [9]	France	572 pregnant female Sprague-Dawley rats, with 20 to 26 animals assigned for each group.	Inhalation exposure of 100, 500, 1000, and 2000 ppm of ethylbenzene, technical xylene, and its isomers from GD 6 until GD 20.	Technical xylene, technical xylene’s individual isomers (o-, m-, and p-xylene), and ethylbenzene.	Inhalational technical xylene exposure of 1000 ppm caused a significant decrease in maternal body weight gain between GD 6 and GD 13. Exposure to 2000 ppm of technical xylene significantly decreased maternal food consumption.
Saillenfait et al. [23]	France	156 pregnant female Sprague-Dawley rats, with 19 to 23 animals assigned for each group.	Inhalation exposure of 250 ppm, 1000 ppm of ethylbenzene and 1000 ppm, 3000 ppm of methyl ketone (individual and combined exposure) from GD 6 until GD 20.	Ethylbenzene and methyl ketone.	Co-exposure of ethylbenzene, and methyl ketone (250 ppm ethylbenzene/3000 ppm methyl ketone) resulted in a significant reduction in maternal body weight and food consumption.
Saillenfait et al. [24]	France	150 pregnant female Sprague-Dawley rats, with 19 to 21 animals assigned for each group.	Inhalation exposure of ethylbenzene (250 ppm, 1000 ppm), toluene (500 ppm, 1500 ppm), and butyl acetate (500 ppm, 1500 ppm) with single or combined exposure from GD 6 until GD 20.	Ethylbenzene, toluene, and butyl acetate.	Exposure to butyl acetate at high concentrations (2000/3000 ppm) reduced maternal weight significantly, but not in combination with ethylbenzene or toluene.
Singh et al. [25]	India	Adult flies and 3rd instar larvae of *D. melanogaster*.	Standard dietary supply for *D. melanogaster* larvae with or without the test chemicals (1,10, 50, 100 mM of test compounds).	Xylene, toluene, and benzene.	Xylene at concentrations of 50 mM and 100 mM delayed the development of adult flies and reduced the number of eggs laid by female *D. melanogaster*.
Malloul et al. [26]	Morocco	21 pregnant female Swiss mice.	Inhalation exposure to 300 ppm and 600 ppm of paint thinner for 30 min (with 5-min interval after 15-min exposure) twice daily during the gestational period.	Paint thinner with major compositions of toluene, xylene, benzene, and dichloromethylene.	Prenatal exposure to 600 ppm of paint thinner caused a significant decline in the maternal weight. Abortion and preterm birth were induced in pregnant rats exposed to 600 ppm of paint thinner.

GD, gestational day; ppm, parts per million.

**Table 6:** Human studies on the toxicity of technical xylene to the female reproductive system from 1990 to 2022.

First Author (Year)	Location	Study Design	Test Populations	Exposure	Organic Solvents Studied	Findings in Relations to Xylene
Cho et al. [27]	China	Cross-sectional human population study	1408 female petrochemical workers.	Inhalation occupational exposure (<1 ppm/day) of organic solvents.	Xylene, toluene, benzene, and styrene.	53% increase (OR, 1.53) in oligomenorrhea from 3 or more years work exposure. 7% percent increase in oligomenorrhea (OR, 1.07) from each additional year of work.
Reutman et al. [28]	United States	Cross-sectional human population study	170 female personnel of the United States’ Air Force between 18 and 42 years old.	Inhalation occupational exposure of aromatic hydrocarbons (exposure levels between 0.8 and 37.3 ppb).	Aromatic hydrocarbons: BTEX. Aliphatic hydrocarbons: hexane, heptane, octane, nonane, decane, and undecane.	Significant reduction in preovulatory LH levels in women with high breath total BTEX (15.8 vs. 22.0 mIU LH/mg creatinine). BTEX were not significantly associated with changes in midluteal estradiol and progesterone.
Aguilera et al. [29]	Spain	Human cohort study	562 pregnant women and their fetuses.	Daily inhalation outdoor exposure to pollutants.	NO_2_ and BTEX.	Exposure to BTEX during early pregnancy caused smaller biparietal diameters in fetuses.
Lupo et al. [30]	United States	Case-control human population study	1108 fetuses of pregnant women delivered with neural tube defects.	Ambient daily exposure of benzene (0.45–7.44 µg/m^3^), toluene (0.31–14.3 µg/m^3^), ethylbenzene (0.05–2.74 µg/m^3^), and xylene 0.36–8.84 µg/m^3^).	BTEX.	There were strong correlations between BTEX and spina bifida, with a significant relationship with benzene. Measured benzene concentrations were between 0.12 (low) and 7.44 (high) µg/m^3^. There were relationships with BTEX and anencephaly.
Ghosh et al. [31]	United States	Human population study	8181 low-birthweight and 370,922 normal-birth-weight children.	Maternal inhalation of traffic air pollutants (0.4 to 3.0 ppb).	BTEX.	Higher odds of giving birth to low weight neonates when exposed to higher concentrations of traffic contaminants during the third trimester.
Santiago et al. [32]	Brazil	Case reports	Two female gas station workers with histories of miscarriages.	Occupational exposure to BTEX at the gas station (8 h/week).	BTEX.	Chronic occupational exposure to BTEX induces chromosomal aberrations linked to reproductive hazards and early pregnancy loss.
Moradi et al. [33]	Iran	Cross-sectional human population study	36 randomly selected beauty salon workers in Tehran metropolitan.	Occupational indoor inhalation exposure of benzene (4.9 µg/m^3^), toluene (20.9 µg/m^3^), ethylbenzene (4.2 µg/m^3^), and xylene (10.1 µg/m^3^).	BTEX.	The prevalence of a menstrual disorder was 47% among exposed workers. Urinary pre-shift concentrations of m-xylene and o-xylene were 99.3 ng/L and 57.6 ng/L, respectively. Urinary post-shift concentrations of m-xylene and o-xylene were 276.3 ng/L and 149.2 ng/L, respectively.
Serrano-Lomelin et al. [34]	Canada	Human population study	333247 singleton livebirths in Canada based on the postal codes from 2006–2012.	Maternal inhalation exposure to industrial air pollutants (1.3% of total mass of air pollutants measured in tons)	Xylene, toluene, and methyl ethyl ketone.	Xylene containing mixtures of industrial pollutants had a significant association with low birth weight (OR 1.16)
Cassidy-Bushrow et al. [35]	United States	Human population cohort study	Women with singleton delivery from 2008–2010.	Inhalation exposure to ambient air pollutants (8 µg/m^3^ BTEX, 18.5 ppb NO_2_, 14.2 µg/m^3^ PM_10_, 10.7 PM_2.5_).	BTEX, NO_2_, PM_10_, PM_2.5_.	Combined with other maternal factors, e.g., age, poverty, and ethnicity, there was a strong association between BTEX exposure and preterm births.
Ding et al. [36]	United States	Cross-sectional human population studies	2432 non-institutionalized women aged 20–49 years on reported use of feminine products.	Exposure to VOCs from feminine products i.e., tampon, sanitary napkin, vaginal douche, feminine spray, powder, and wipes.	Xylene, toluene, ethylbenzene, bromodichloromethane, chloroform, dibromodichloromethane, and dichlorobenzene.	The use of feminine powder was significantly associated with a 35.6% higher whole blood concentration of ethylbenzene (35.6 ng/mL of blood), compared with the never users. The use of feminine powder, vaginal douche, feminine spray, and wipes was significantly higher among non-Hispanic black females.

OR, odd ratio; LH, luteinizing hormone; BTEX, benzene toluene ethylbenzene xylene; ppb, parts per billion; ppm, parts per million; VOC, volatile organic compound; NO_2_, nitrogen dioxide; PM, particulate matter.

## Data Availability

Data are available in the article.
